# Sorbic acid stress activates the *Candida glabrata* high osmolarity glycerol MAP kinase pathway

**DOI:** 10.3389/fmicb.2013.00350

**Published:** 2013-11-26

**Authors:** Zeljkica Jandric, Christa Gregori, Eva Klopf, Martin Radolf, Christoph Schüller

**Affiliations:** ^1^Department of Applied Genetics and Cell Biology (DAGZ), University of Natural Resources and Life SciencesVienna, Austria; ^2^MFPL, Department of Medical Biochemistry, Medical University of ViennaVienna, Austria; ^3^Research Institute of Molecular PathologyVienna, Austria

**Keywords:** sorbic acid, HOG pathway, Candida glabrata, stress response, Fungal pathogen

## Abstract

Weak organic acids such as sorbic acid are important food preservatives and powerful fungistatic agents. These compounds accumulate in the cytosol and disturb the cellular pH and energy homeostasis. *Candida glabrata* is in many aspects similar to *Saccharomyces cerevisiae*. However, with regard to confrontation to sorbic acid, two of the principal response pathways behave differently in *C. glabrata*. In yeast, sorbic acid stress causes activation of many genes via the transcription factors Msn2 and Msn4. The *C. glabrata* homologs CgMsn2 and CgMsn4 are apparently not activated by sorbic acid. In contrast, in *C. glabrata* the high osmolarity glycerol (HOG) pathway is activated by sorbic acid. Here we show that the MAP kinase of the HOG pathway, CgHog1, becomes phosphorylated and has a function for weak acid stress resistance. Transcript profiling of weak acid treated *C. glabrata* cells suggests a broad and very similar response pattern of cells lacking CgHog1 compared to wild type which is over lapping with but distinct from *S. cerevisiae*. The *PDR12* gene was the highest induced gene in both species and it required CgHog1 for full expression. Our results support flexibility of the response cues for general stress signaling pathways, even between closely related yeasts, and functional extension of a specific response pathway.

## Introduction

Candida species cause mucosal as well as disseminated infections in humans. Many women experience Candida vulvovaginitis, with recurring infections (Buitron Garcia-Figueroa et al., 2009). *Candida albicans* is the leading cause of such infections, with *Candida glabrata* accounting for about 20% (Schmalreck et al., 2012). Compared to *C. albicans*, *C. glabrata* is more similar to *Saccharomyces cerevisiae* (Dujon, 2010) and might be regarded as a Saccharomycete with adaptations to mammalian commensalism. Striking adaptations are the optimal growth temperature of 37°C, its short division time of about an hour at this temperature (Roetzer et al., 2011), and the ability to adhere to various surfaces due to a number of adhesins (Domergue et al., 2005). *C. glabrata* has a characteristic high resistance to certain common antifungal drugs such as azoles (Cross et al., 2000; Pfaller and Diekema, 2007). All *C. glabrata* isolates are haploid suggesting an absent or greatly reduced sexual cycle. The inability to differentiate into spores as a resistant cell type is perhaps outweighed by high persistence due to stress and drought resistance (Berila and Subik, 2010).

Adjustment of metabolism and cell cycle to changing environmental conditions is essential for microbial organisms. Evidently, a pathogen has to gather specific information about its host environment (Shapiro et al., 2011). Fungi communicate and respond to their environment with an array of signaling components such as MAP kinase pathways (Chen and Thorner, 2007), dedicated transcription factors such as the heat shock transcription factor Hsf1 or the copper responsive factor Ace2, and conserved signaling pathways such as the protein kinase A (PKA), target of rapamycin (TOR), DNA damage responsive pathways and many more. Fungal cells use this information to make decisions to pass cell division checkpoints and for channeling of resources toward defence or growth (Zakrzewska et al., 2011). Thus, environmental response signaling pathways are one putative Achilles' heel for intervention with fungal growth and establishment in environmental niches provided by the host. In fact, some combinations of stress types are efficiently preventing fungal growth (Kaloriti et al., 2012).

During antifungal therapy mutations arise in special genes such as drug efflux pumps and their regulators due to selective pressure (Ferrari et al., 2011a,b). However, the clue to the success of *C. glabrata* lies possibly in its ability to persist. The intrinsic stress resistance of *C. glabrata* likely has a multigenic basis and a dynamic component which can be established by external conditions and might even be propagated by epigenetic mechanisms. The transient aspect of resistance traits of clinical isolates has not been explored systematically.

The well-characterized high-osmolarity glycerol (HOG) pathway is essential for yeast survival under-high osmolarity conditions, since it triggers adaptation through intracellular accumulation of glycerol as the adaptive osmolytes to reestablish the balance of water and ion concentration (De Nadal and Posas, 2010). Yeast Hog1 becomes activated by dual phosphorylation and translocates from the cytosol to the nucleus to change gene expression patterns. In addition, Hog1 has cytosolic targets such as the Fps1 aquaglyceroporin (Thorsen et al., 2006; Mollapour and Piper, 2007). Stress activated protein kinase networks are well conserved across kingdoms and orthologs of Hog1 are present in other fungi (Nikolaou et al., 2009). However, the detailed action of Hog1 in *C. glabrata* and its downstream targets have not been investigated in depth so far. Hog1 is related to the p38 MAP kinase of higher eukaryotes. There, this kinase has a broader role as initially described for *S. cerevisiae* Hog1 which is mainly responsive to osmolarity changes. However, other stress types such as oxidative stress as well as exposure to methylglyoxal (Aguilera et al., 2005) or acetic acid (Mollapour and Piper, 2006) activate this pathway. In *C. albicans*, Hog1 becomes activated by oxidative stress and Cadmium exposure (Enjalbert et al., 2006; Yin et al., 2009). In yeast and *C. glabrata*, the HOG signaling pathway is required for osmotic stress response and oxidative stress (Kaloriti et al., 2012). In contrast to yeast, however, CgPbs2 (the MAPKK) mutants display hypersensitivity to weak organic acids (Gregori et al., 2007b). These results would suggest that CgHOG pathway compared to ScHog1 has acquired or retained other functions.

Short chain carboxylic acids or weak organic acids are in widespread use as preservatives for food and feed (Plumed-Ferrer and Von Wright, 2011). A recent excellent review covers the effects of weak acids on yeasts (Piper, 2011). Weak acid response at the transcriptional level is mediated by the zinc cluster transcription factor War1 (weak acid response 1) in *S.cerevisiae, C. albicans*, and *C. glabrata* (Kren et al., 2003; Lebel et al., 2006; Mundy and Cormack, 2009). Remarkably, War1 is not conserved in intrinsically highly weak acid resistant food spoiling Zygomycetes (Mollapour et al., 2008). In *S. cerevisiae* this factor triggers expression of a small regulon consisting of an ABC (ATP binding cassette) transporter gene *PDR12* which is required for weak acid ion efflux as well as an ammonia transporter *FUN34* (Kren et al., 2003; Schüller et al., 2004; Gregori et al., 2007a). Strikingly, activation of *PDR12* transcription is sufficient for weak acid resistance (Schüller et al., 2004). Activation of War1 by weak acids is possibly rather direct, (e.g., interaction with the acid or a metabolite) since no regulatory factors upstream of War1 have been identified to date by genetic means. Attempts to assign a regulatory function to weak acid induced changes of the War1 phosphorylation status, which results in a robust migration difference, were unsuccessful (Frohner et al., 2010; Mollapour and Piper, 2012). It has been suggested that War1 becomes directly activated by weak acids triggering conformational changes (Gregori et al., 2008). In addition, adaptation to weak acids also requires Haa1, a further weak acid inducible transcription factor *S. cerevisiae* (Fernandes et al., 2005).

*S. cerevisiae* cells treated with weak organic acids accumulate the transcription factors Msn2 and Msn4 rapidly in the nucleus and activate a relative large regulon of generic stress responsive genes (Schüller et al., 2004). Remarkably, induction of the majority of sorbate-induced genes required Msn2/4, however, weak organic acid tolerance was unaffected by a lack of Msn2/4. Strikingly, CgMsn2 did not accumulate in *C. glabrata* cells exposed to weak acids (sorbate and propionate). However, CgMsn2 expressed in yeast cells showed similar nuclear accumulation as ScMsn2 (Roetzer et al., 2008). Thus, *C. glabrata* either lacks a pathway which is operative in *S. cerevisiae* or responds differently to weak acids. For example, different cellular acidification of weak acid exposed *S. cerevisiae* or *C. glabrata* cells could play a role (Ullah et al., 2013b). Alternatively, weak acids might affect growth and thus Msn2/4 activation indirectly (Zakrzewska et al., 2011). Weak acids induce expression of the *C glabrata* adhesin Epa6 via an Msn2/4 and War1 independent mechanism leading to enhance adherence. This is remarkable because use of weak acids is widespread as preservative (not as the active ingredient) in over-the-counter vaginal products (Mundy and Cormack, 2009).

We report the generation of a *C. glabrata* strain deleted for *HOG1*. We find that sorbic acid stress causes a robust high level phosphorylation of CgHog1 coinciding with an enrichment in the nucleus. Moreover, we show that CgHog1 is required for sorbic acid resistance of *C. glabrata*.

## Materials and methods

For gene disruption of *HOG1* in the BG14 background, we used the *SAT1* nourseothricin resistance marker cassette amplified from plasmid pSFS2 (Reuss et al., 2004). Via three-way PCR a knockout cassette with long homologous flanking regions was created and integrated into the BG14 strain. Correct insertion of the cassette was verified by genomic PCR. Primer sequences: Hog1–1 5′GGC TAC TAA TGA AGA GTT CAT AAG, Hog1–2 5′cac ggc gcg cct agc agc ggC TAC TCC TGC TGA GTG AAC G, Hog1–3 5′gtc agc ggc cgc atc cct gcC AGA GGC AAA GTT TGA CTG G, Hog1–4 5′CAC TGC TTG ATT AGC ATA CTC. To determine susceptibilities to osmostress and sorbic acid exponentially growing cultures were adjusted to an optical density at 600 nm (OD_600_) of 0.1 and diluted 1:10, 1:100, and 1:1,000. Equal volumes of serial dilutions were spotted onto YPD (pH 4.5, adjusted with HCl) plates containing various concentrations of NaCl and potassium sorbate. Plates were incubated at 30°C for 36–48 h.

### Hog1 phosphorylation analysis

The phosphorylated Hog1 isoforms were detected using two different anti-phospho-p38 MAPK antibodies as indicated in Figure 3. Phospho-p38 MAPK (Thr180/Tyr182) (Cell Signaling Technologies #9211) recognizes either Thr180 or Tyr182 phosphorylated species while antibody ab4822 (Abcam) recognizes double Y182 + T180 phosphorylated species, according to the manufacturers. Cells were grown to OD_600_ of 1 treated for 40 min with potassium sorbate, harvested and frozen. TCA extracts were prepared, and cell lysates equivalent to 0.5 OD_600_ unit were fractionated by 10% sodium dodecyl sulfate-polyacrylamide gel electrophoresis (10% SDS-PAGE) and transferred to nitrocellulose membranes. ScPgk1 antibodies were used to detect CgPgk1 as loading control as described (Gregori et al., 2007a), or a cross reaction served as loading control. The *CgHOG1* open reading frame was cloned into the unique BamHI site of vector pGRB2.2 using sites introduced by PCR immediately upstream of the start codon and replacing the stop codon (Mumberg et al., 1995; Frieman et al., 2002) and fused to yEGFP originating from pKT128 (Sheff and Thorn, 2004) introduced with a unique EcoRI in front of the start codon and a SalI site after the stop codon.

### Microscopy

Cells expressing GFP tagged variants of CgHog1 were grown to mid-exponential phase. Appropriate cultures were visualized live with a CELL R system (Olympus, Japan) and detected with an Orca R2 camera (Hamamatsu Photonics K.K. Japan). Quantification of the fluorescence intensity was done with ImageJ. The background subtracted signal ratio of cytosol to nucleus was determined comparing the fluorescence signals obtained from both compartments during 10 min 10 mM of sorbic acid stress, and non-stress conditions.

### Expression profiling

For microarrays, BG14 and BG14*hog1*Δ strains were grown for 4 generations in 50 ml cultures in YPD at 30°C to OD_600 nm_ of about 1 before potassium sorbate was added to a final concentration of 20 mM. Three biological replicates were generated. After 20 min cells were harvested and immediately frozen. RNAs were prepared as described earlier (Klopf et al., 2009)1 μg of total RNA was used for labeling reaction (Agilent Quick Amp Labeling Kit, two-color, Cat.Nr. 5190–0444). The standard Agilent Protocol for one-color labeling was used (G4140–90041). 325 ng of Cy3 labeled cRNA were hybridized to the custom *C. glabrata* GE 8 × 15 K arrays [Agilent eArray Design 017617; GPL10713 (Ferrari et al., 2011b)]. Samples were hybridized for 17 h at 65°C, 10 rpm. Agilent G2505C Microarray Scanner System was used to scan the arrays. The Agilent Feature Extraction program (Version FE 10.5.1.1) was used to analyze the array images. Values were normalized with quantile scale transformation at Babelomics (http://babelomics.bioinfo.cipf.es) using the Bioconductor affy package. Duplicate features were averaged. Features with gProcessedSignal values >8 (log2) (equals ~10 fold signal over background) were retained and per condition average of 2 and 3 values further analyzed. GO terms enriched in selected sets were extracted via String (string-db.org) and GOTermfinder (yeastgenome.org). Analyzed with Cluster analysis, using cluster3 and visualized with TreeView (Saldanha, 2004) (http://jtreeview.sourceforge.net). TreeView files corresponding to the figures and the raw data are supplied (Supplematary Datafile1). Data have been submitted to GEO (accession No. GSE52382).

qRT-PCR measurement of *PDR12* transcript levels of cells treated with 20 mM sorbic acid for 20 min at neutral pH. Poly T anchored cDNA was synthesized by using Revert Aid Reverse Transcriptase (Thermo Scientific). *PDR12* cDNA was detected using RT-Primers within the coding sequence Pdr12-RT (for.) GGAAAGGAAGGATGATGCAGA and Pdr12-RT (rev.) CTGGCCATGGACTCCAATCTT. *ACT1* was used as a stress unresponsive internal control and amplified via Act1-RT (for.) ATCGTTTCCCCCTTTGCCAC and Act1-RT (rev.) TGCCCACCACTCCTAACTCA.

## Results

**Figure 1:**
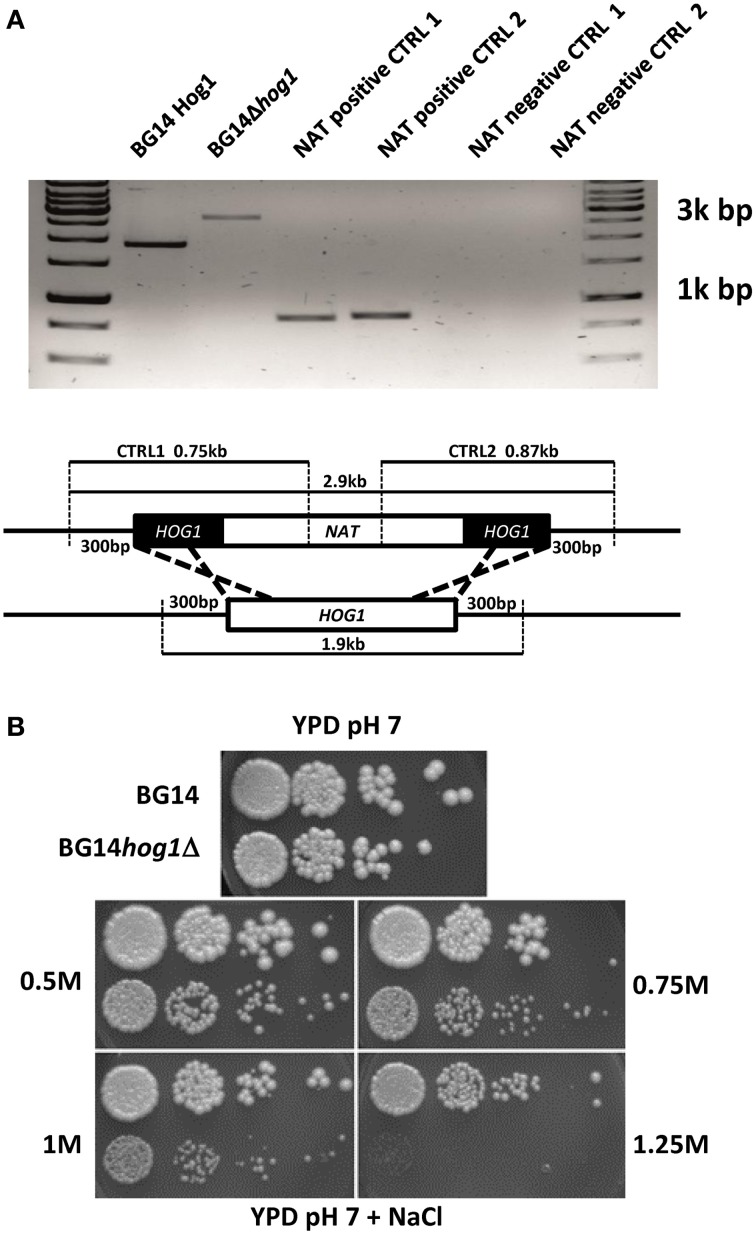
**(A)** Confirmation of the integration of the marker cassette in to the Cg*HOG1* locus. The genomic locus and primer binding sites is indicated. **(B)** *C. glabrata* Hog1 is required for osmotic stress tolerance. BG14, BG14*hog1*Δ, and BG14 carrying pHog1-GFP were diluted and spotted on YPD containing the indicated amount of NaCl. Growth was recorded after about 36 h.

**Figure 2:**
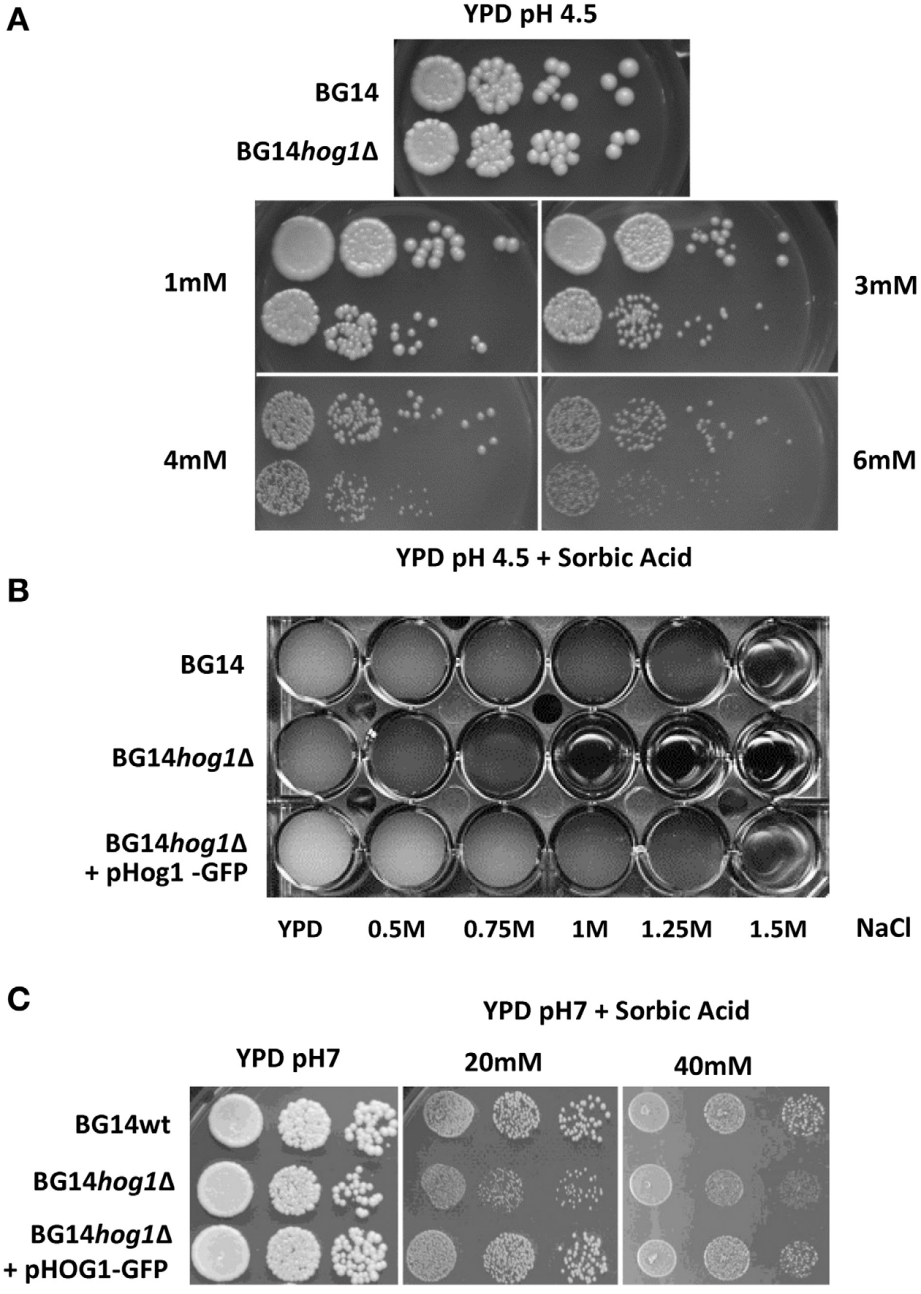
**(A)** *C. glabrata* mutants deleted for *HOG1* display sensitivity to weak acids. Cultures of the *C. glabrata* strains BG14 and BG14*hog1*Δ, were diluted and spotted onto rich medium containing the indicated amounts of sorbic acid at pH 4.5. Growth was recorded after about 36 h for the control and 48 for weak acid stress. **(B)** CgHog1-GFP complements the osmotic stress sensitivity of the BG14*hog1*Δ strain. Cultures of BG14, BG14*hog1*Δ and BG14*hog1*Δ transformed with CgHog1-GFP were diluted to OD_600_ of 0.01 in YPD and exposed to a range of NaCl concentrations. Growth was recorded after 24 h. **(C)** CgHog1 is required for growth under sorbic acid stress. Cultures of wild type and BG14*hog1*Δ cells with and without CgHog1-GFP were diluted and spotted onto medium containing the indicated amounts of sorbic acid at neutral pH. CgHog1-GFP complements the growth defect of BG14*hog1*Δ under sorbic acid stress. Growth was recorded after 36 h of incubation.

The *C. glabrata HOG1* (CAGL0M11748g) gene was deleted in the BG14 background (Mundy and Cormack, 2009) by homologous recombination and replaced with the nourseothricin resistance gene. It was not possible to recover mutants with eliminated open reading frame due to high background of false positives caused by frequent random integration of the corresponding disruption cassette. We were successful with the deletion of the region between ORF base pair positions 402–960 eliminating the coding sequence for the active site of the kinase domain. The correct integration of the cassette was verified by genomic PCR (Figure 1A). The Cg*HOG1* deleted strain, BG14*hog1*Δ, was sensitive to high osmolarity conditions higher than 0.5 M NaCl (Figure 1B). The *C. glabrata* mutant lacking Hog1 could tolerate higher salt concentration than the corresponding *S. cerevisiae* mutant strain. In addition to the expected phenotype of high osmolarity sensitivity, BG14*hog1*Δ, was sensitive to sorbic acid stress (Figure 2A). We found reduced growth on pH4.5 medium containing 1 mM sorbic acid and severely reduced growth with 3 mM, as observed earlier (Gregori et al., 2007b). At neutral pH, concentrations above 20 mM sorbic acid were required to inhibit growth (Figure 2C). The overall sensitivity of the *HOG1* deleted strain was similar to the mutant lacking the MAPKK Pbs2 (Gregori et al., 2007b). Notably, *S. cerevisiae* strains lacking Hog1 are not sensitive to sorbic acid.

**Figure 3:**
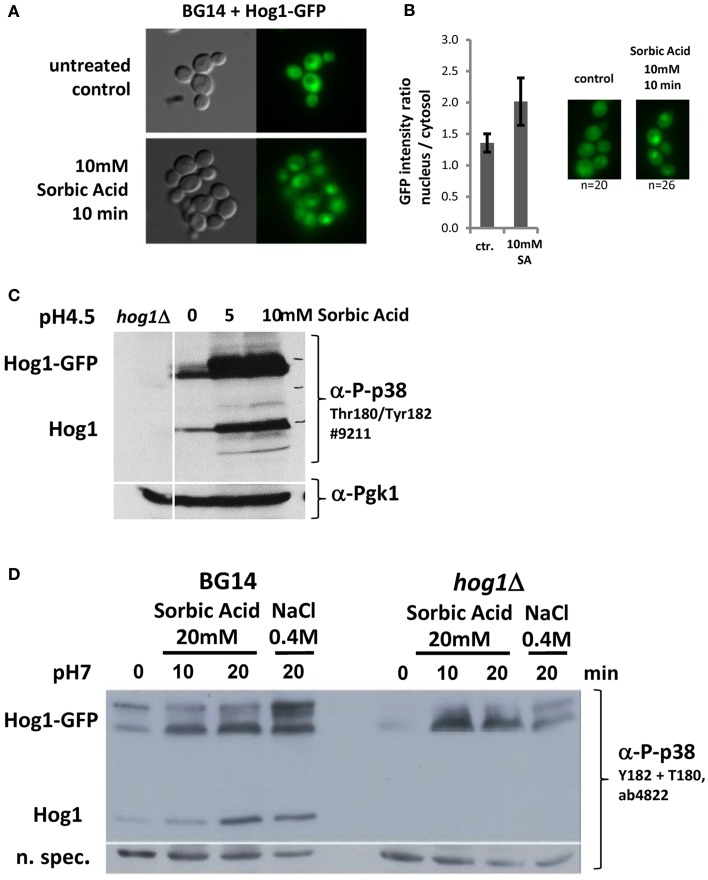
**(A)** CgHog1 accumulation increases in the nucleus of sorbic acid stressed cells. Life Microscopy of BG14 expressing CgHog1-GFP without stress (upper panel) or under Sorbic acid exposure (10 min, 10 mM, lower panel). **(B)** Nuclear accumulation of Hog1 upon stress induction results in a higher fluorescence signal and increases the nucleus to cytosol signal ratio. Fluorescence intensity ratio, nucleus to cytosol, in unstressed (*n* = 20) and stressed (*n* = 26) cells. **(C)** CgHog1 and CgHog1-GFP phosphorylation increases in sorbate treated cells. Cultures of BG14, BG14*hog1*Δ, and BG14 carrying pHog1-GFP were grown in YPD (pH 4.5) to the early-exponential-growth phase before 5 or 10 mM Sorbic acid was added. Samples were harvested after 20 min. Detection was carried out using polyclonal anti-phospho-p38 MAPK or anti-Pgk1 antibodies. **(D)** Dual phosphorylation of Hog1 upon sorbic acid and osmotic stress. Cultures of BG14, BG14*hog1*Δ, and BG14 carrying pHog1-GFP were grown in YPD (pH7) to the early-exponential-growth phase. Sorbic acid was added to a final concentration of 20 mM, samples were taken after 10 and 20 min. Osmotic stress (0.4 M NaCl) for 10min was used to compare to a standard induced Hog1 phosphorylation pattern.

We next investigated if CgHog1 is activated due to weak acid stress exposure. Activation of Hog1 by phosphorylation of the activation loop residues by the MAPKK Pbs2 causes its rapid translocation into the nucleus. A fusion gene of GFP to the Cg*HOG1* open reading frame was created and expressed from a plasmid. The CgHog1-GFP fusion construct complemented the deleted allele (Figures 2B,C). The fluorescence signal of the CgHog1-GFP construct showed a substantial nuclear accumulation in unstressed cells. A similar partial nuclear accumulation of Hog1-GFP in unstressed cells has also been observed in our lab in another *C. glabrata* strain background (CBS138). Partial nuclear localization CgHog1-GFP in unstressed cells is possibly due to a certain level of basal activation of CgHog1 in BG14 cells (Figures 3C,D). Despite an apparent partial activation in unstressed cells, exposure to sorbic acid led to an enhanced staining of nuclei by Hog1-GFP within short time. The microscopy pictures shown in Figure 3 were scanned after about 10 min. However, Hog1-GFP nuclear accumulation was visible immediately after addition of sorbic acid.

To support a direct role of the HOG pathway during weak acid stress we determined the CgHog1 phosphorylation status. We analyzed phosphorylation of the CgHog1 activation loop with antibodies recognizing the p38 mono and double phosphorylated TEY motif. Interestingly, and different from *S. cerevisiae*, we found signals indicating phosphorylation and thus active CgHog1 in unstressed cells (Figures 3C,D). CgHog1 phosphorylation level increases in cells treated with 5 and 10 mM sorbic acid for 40 min at pH4.5 (Figure 3C). Both the endogenous CgHog1 and the plasmid encoded CgHog1-GFP fusion protein showed similar changes of phosphorylation levels upon treatment with sorbic acid. The signal originating from CgHog1 is identified by testing a BG14*hog1*Δ strain without GFP plasmid (Figure 3D). Interestingly, the GFP fusion protein splits into two species of which the slower migrating form shows more dynamic changes of phosphorylation. The signals obtained suggest that two pools of CgHog1 might exist in *C. glabrata* which are perhaps not separated at the level of the wild type size protein. One is apparently constitutively phosphorylated and the other shows dynamic changes. We repeated the experiment with an antibody recognizing the double phosphorylated form to confirm that the observations are not due to a mono-phosphorylated TEY, and thus likely inactive CgHog1. In addition we exposed the cells at neutral pH to sorbic acid to avoid additional stress. We detected an increase of CgHog1 and of CgHog1-GFP phosphorylation to a comparable level in the osmotic and sorbic acid stressed case (Figure 3D).

**Figure 4:**
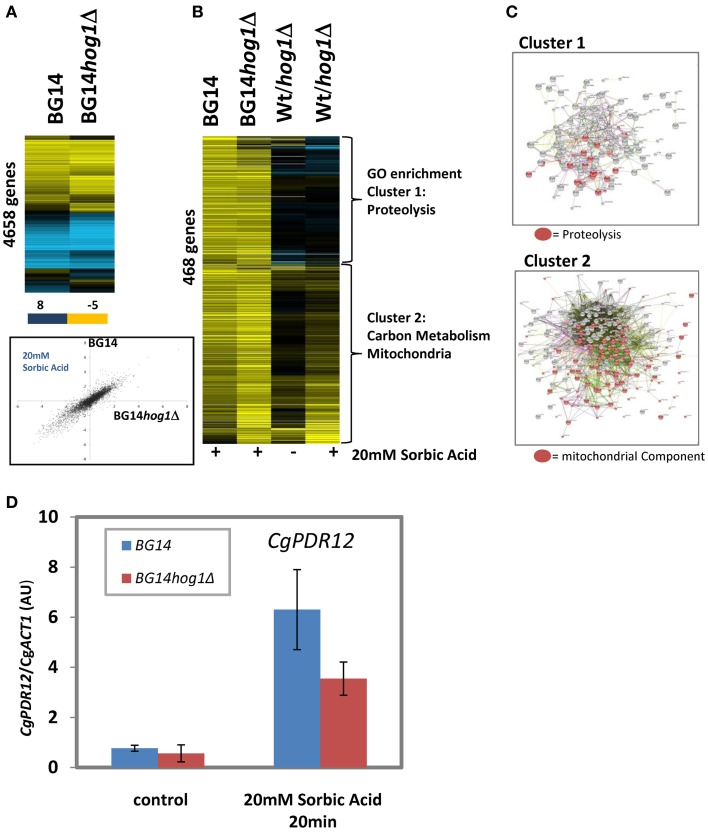
**Differentially expressed genes in BG14 and BG14*hog1*Δ cells, treated with 20 mM Sorbic acid for 20 min. (A)** Overall comparison of gene expression changes in wild type and mutant. Scatter Plot of sorbate treated wild type vs. sorbate treated *hog1*Δ. **(B)** Clustering of selected genes with higher than 2-fold change of transcript level isolates two prominent clusters enriched in the indicated GO terms. **(C)** String connection maps of cluster one and two. **(D)** *CgPDR12* mRNA quantification after sorbic acid stress (20 mM, pH7, 20 min).

The consequence of Hog1 activation in *C. albicans* and *S. cerevisiae* is, apart from changes of the activity of cytosolic targets (Thorsen et al., 2006; Mollapour and Piper, 2007), nuclear translocation and a dramatic change of gene expression mediated by a set of transcription factors. We were interested to which extend CgHog1 is influencing transcriptional patterns of sorbic acid stressed *C. glabrata* cells. We used single color arrays as described in materials and methods and report the results from a 20 min treatment with 20 mM Sorbate at neutral pH of BG14 and BG14*hog1*Δ. The overall response pattern of repressed and induced genes in was very similar between mutant and wild type as seen in a clustered presentation and a scatter plot (pearson correlation coefficient *r* = 0.84 of genes in Figure 4A). The highest induced genes in both wild type and mutant were Cg*PDR12*, Cg*BTN1* (possible role in mediating pH homeostasis), Cg*HSP42* (a small heat shock protein with chaperone activity), Cg*DCS2* [m(7)GpppX pyrophosphatase regulator protein]. Further differentially regulated genes are listed in (Supplemantary DataSheet1/Top_changed). The repressed genes were enriched in protein synthesis related functions. We selected 466 genes with induced expression (>2.25 fold) in wild type or mutant (Figures 4B,C). These can be divided into two clusters. Cluster one is enriched in genes with functions in proteolysis, autophagy and mainly cytosolic components. GO terms are reported in supplementary material (Supplemantary DataSheet1). For a small number of genes of this cluster, the fold increase of was reduced by a factor of more than 2 in absence of CgHog1 (Supp DataSheet1/Top_changed; *CAGL0L05016g STB6, CAGL0K07205g* (unknown)*, CAGL0C02321g PHM8, CAGL0F01111g OPI10, CAGL0B00858g STE50, CAGL0K07590g, MYO3*). These genes have striking relations to starvation and stress resistance. Phm8 is a Lysophosphatidic acid (LPA) phosphatase active in response to phosphate starvation. Ste50 is involved in mating, filamentous growth and osmotolerance. If these or other transcripts changed in the mutant contribute to the sorbic acid sensitivity phenotype remains to be shown. Expression levels of transcription factors with a function for to stress response are rarely regulated by stress due to their required latent presence. Interestingly, *CgMSN4* is induced 5-fold (and higher in the mutant), whereas Cg*COM2* has a robust 2.6-fold increase of expression. Yer130C (designated *COM2* for Cousin Of Msn2) is a Zn-finger protein similar to ScMsn2/4 and *C. albicans* Mnl1 and interestingly its expression is induced by acetic acid stress (Mira et al., 2011). Therefore, Yer130C/Com2 might be connected to weak acid stress in both organisms.

Cluster two comprises the remaining genes of this set and is strikingly enriched in mitochondrial functions and carbon metabolism (Supplementary GO data in DataSheet1). Furthermore, cluster two is enriched in genes with lower expression levels in unstressed mutant cells. This result is indicated by String (http://string-db.org) network analysis. Comparison to *S. cerevisiae* sorbic acid stress data (Schüller et al., 2004; SuppTable1: SCvsCG_Sorbat) results in a mixed picture of a surprisingly weak correlation coefficient (*r* = 0.25), however, some characteristic similarities such as *PDR12* as the most highly induced gene exist. Inspection of the pattern of genes induced in both organisms raises a suspicion of less pronounced increase of *S. cerevisiae* Msn2/4 orthologous target genes in *C. glabrata*. However, part of the difference is of technical nature.

Failure to express the *CgPDR12* gene could contribute to the sensitivity of the CgHog1 deletion mutant. The *S. cerevisiae PDR12* gene confers most of weak acid resistance (Schüller et al., 2004). In our array data the *PDR12* gene changes more than 160-fold in the wild type and 130-fold in the mutant. We confirmed this by RT-qPCR in two independent biological replicas and found a fold change of >10-fold (due to higher unstressed background signal) and a similarly reduced level in the BG14*hog1*Δ strain (Figure 4D). Whether this mRNA level difference may account for the sorbic acid sensitivity phenotype remains to be shown.

## Discussion

We report an initial characterization of the *C. glabrata* Hog1 MAP kinase and show activation of the HOG pathway by weak acids in this yeast. Weak acids like sorbic or benzoic acid traverse the plasma membrane in the protonated form and dissociate in the cytosol (Piper, 2011). The consequences of this influx are manifold. Yeast cells respond to weak acid stress with dedicated defense mechanisms like the War1-Pdr12 system but also to the indirect damage of weak acid accumulation. Previously, the *C. glabrata* HOG pathway has been shown to be involved in weak acid and acetate resistance (Gregori et al., 2007a). Acetate activates the pathway in both yeasts at concentrations above 50 mM. We show here that weak acid treatment causes a rapid increase of dually phosphorylated CgHog1 and thus signaling through this pathway. The sorbic acid stress sensitivity of the CgPbs2 mutant further indicates activation of the *C. glabrata* MAP kinase cascade but the sensing mechanism upstream is yet unknown (Gregori et al., 2007a). As an additional striking difference to *S. cerevisiae*, we observe a relatively high phosphorylation level of the CgHog1 kinase in unstressed cells. In contrast to ScHog1, and possibly connected to its basal phosphorylation, we find a partial accumulation of CgHog1 in the nucleus in unstressed cells. This could have consequences on the level of gene expression since our microarray experiment shows differences between the transcript patterns of unstressed deletion mutant and wild type. Basal expression of many genes linked to mitochondrial function and carbon catabolism is reduced in the mutant. The relevance of this observation to weak acid resistance or other phenotypes e.g. *C. glabrata* virulence remains to be investigated. In fact, the *in vivo* function of this characteristic might not at all be connected to the host environment. A constitutive partially active HOG pathway, which is not well tolerated by *S. cerevisiae*, could prepare *C. glabrata* cells for water deprivation and starvation *in vitro* (outside the host) and thus enhance persistence. A role for *S. cerevisiae* Hog1 for the re-entry into the cell cycle after starvation induced resting has been reported (Escote et al., 2011). Strikingly, Hog1 rapidly enters the nucleus at the time of resumption of growth in absence of osmotic stress. Since p38 shows similar response, this might represent a conserved SAPK-dependent response for cells leaving a resting state (Escote et al., 2011). ScHog1 activates some of its many target genes directly by interacting with Msn2 and other transcription factors. In this way ScHog1 becomes tethered to the respective target promoters and contributes substantially to gene regulation. CgMsn2 and CgMsn4 do not translocate into the nucleus in weak acid stressed *C. glabrata* cells, suggesting that they are presumably not involved (Roetzer et al., 2008). The cause for this difference to *S.cerevisiae* Msn2 is unclear, however, is not related to difference in intracellular acidification of sorbate exposed cells (Ullah et al., 2013b). Nevertheless, in *C. glabrata* a similar large number of genes change expression upon Sorbate treatment suggesting other factors substitute for their function. The sensitivity of the CgHog1 deletion mutant could originate from failure to express the *CgPDR12* gene. We think that this is not likely the case. Our gene expression dataset shows a large change of Cg*PDR12* transcript level. Cells lacking Hog1 had 2/3^rd^ of wild type *CgPDR12* mRNA level and thus the HOG pathway does not contribute substantial to transcriptional regulation of the weak acid anion pump. Whether this mRNA level difference does cause part of the sensitivity phenotype remains to be shown. CgHog1 could also be involved in tuning membrane transport components and thus influence weak acid induced ATP expense, which has been reported recently to be regulated to prevent futile cycling (Ullah et al., 2013a).

Taken together, we show that weak acids trigger the *C. glabrata* HOG response pathway leading to MAP kinase activation and nuclear accumulation. Whether this interesting change of response pattern of the HOG pathway has relevance for virulence or persistence of *C. glabrata* requires further investigation.

### Conflict of interest statement

The authors declare that the research was conducted in the absence of any commercial or financial relationships that could be construed as a potential conflict of interest.
